# Highlights and recent developments in allergic diseases in EAACI journals (2019)

**DOI:** 10.1186/s13601-020-00366-3

**Published:** 2020-12-03

**Authors:** J. Bousquet, C. E. Grattan, C. A. Akdis, P. A. Eigenmann, K. Hoffmann-Sommergruber, I. Agache, M. Jutel

**Affiliations:** 1 MACVIA-France, Montpellier, France; 2 Swiss Institute of Allergy and Asthma Research (SIAF), University Zurich, Davos, Switzerland; 3 St John’s Institute of Dermatology, Guy’s Hospital, London, UK; 4 Pediatric Allergy Unit, University Hospitals of Geneva, Geneva, Switzerland; 5 Depart of Pathophysiology and Allergy Research, Medical University of Vienna, Vienna, Austria; 6 Transylvania University Brasov, Brasov, Romania; 7 Department of Clinical Immunology, Wrocław Medical University, Wrocław, Poland; 8 ALL-MED Medical Research Institute, Wrocław, Poland; 9 CHRU Arnaud de Villeneuve, 371 Avenue du Doyen Gaston Giraud, 34295 Montpellier Cedex 5, France

**Keywords:** Asthma, Rhinitis, Atopic dermatitis, Urticaria, Anaphylaxis, Food allergy, Angioedema, Allergens, Immunotherapy, EAACI

## Abstract

The European Academy of Allergy and Clinical Immunology (EAACI) owns three journals: Allergy, Pediatric Allergy and Immunology and Clinical and Translational Allergy. One of the major goals of EAACI is to support health promotion in which prevention of allergy and asthma plays a critical role and to disseminate the knowledge of allergy to all stakeholders including the EAACI junior members. There was substantial progress in 2019 in the identification of basic mechanisms of allergic and respiratory disease and the translation of these mechanisms into clinics. Better understanding of molecular and cellular mechanisms, efforts for the development of biomarkers for disease prediction, novel prevention and intervention studies, elucidation of mechanisms of multimorbidities, entrance of new drugs in the clinics as well as recently completed phase three clinical studies and publication of a large number of allergen immunotherapy studies and meta-analyses have been the highlights of the last year.

## Introduction

The European Academy of Allergy and Clinical Immunology (EAACI) has three official journals: Allergy, Pediatric Allergy and Immunology and Clinical and Translational Allergy. One of the major goals of EAACI is to support health promotion in which prevention and control of allergy plays a critical role and to disseminate the knowledge of allergy to all stakeholders including the EAACI junior members.

The EAACI journals have reported on all fields of allergy and related diseases in 2018 and 2019 [1–4]. This paper summarises some of the achievements of 2019. Several EAACI Position Papers have been published in 2019 [5–15].

## Allergic diseases

Der p 24 is a major *Dermatophagoides pteronyssinus* (HDM) allergen with strong IgE binding activity via an immunodominant IgE epitope in the N-terminal 32-residue region, which triggers IgE production in vivo [16]. The identified Der p 24 epitope may support HDM allergy diagnosis and treatment. Major mite allergens from European standardized commercial extracts for in vivo diagnosis were evaluated [17]. Certain natural *Dermatophagoides pteronyssinus* and *Blomia tropicalis* extracts lack important allergens showing a considerable variability in composition and content. There is a need to improve the quality and consistency of these extracts to achieve a more precise diagnosis and treatment of house dust mite allergy. Skin prick tests (SPTs) are essential for the diagnosis of IgE-mediated allergy and are influenced by extract quality, biological potency and concentration of allergen. An open multicentre study with 431 patients assessed sensitivity and specificity of standardised allergen extracts in skin prick test for diagnoses of IgE-mediated respiratory allergies [18]. In the FRAAT birth cohort in Colombia [19], IgE sensitization is different from that in developing countries and is influenced by the hygienic conditions influence sensitization to *Blomia tropicalis* allergenic components. Clinical case history, previous SPT and specific IgE measurement could all be used as reference methods for the assessment of sensitivity/specificity of SPT solutions. Five to ten percent of the population in affluent countries are allergic to dogs. Diagnosis and treatment is based on allergen extracts from natural sources where composition and concentration are poorly defined. Six allergens from dog extracts were studied [20]. There is a great variation of dog allergens in natural extracts raising questions of source, sampling, processing and ultimately of standardization, and minimum allergen levels for accurate diagnosis and treatment.

Preterm influences have an impact on allergic diseases. In utero exposure to chemicals used in personal care products and plastics may contribute to increase in allergic diseases. Urinary concentrations of eight phthalate metabolites and bisphenol A were quantified in mothers twice during pregnancy in 1999–2000 in Salinas, California [21]. Monocarboxyisooctyl phthalate was associated with lower respiratory health after controlling for related chemical exposure, which suggests that confounding by multiple chemical exposures should be considered in future research. The immune system of preterm infants is immature, being a significant cause of morbidity and mortality, particularly in the preterm infant. Oropharyngeal colostrum administration is safe in preterm neonates and improves their immunologic profile, showing a potential role as an immunomodulatory agent [22]. In the European birth cohort PASTURE, there was an association between exposure to antibiotics and allergies, mainly atopic dermatitis and food allergy within the first year of life, after prenatal exposure, and atopic dermatitis and asthma after post-natal exposure to antibiotics in children born in rural settings [23]. Fetal growth restriction is associated with higher risks of childhood respiratory morbidity. Fetal blood flow adaptations might contribute to these associations. In the Generation R study, it was shown that third-trimester fetal blood flow patterns might be related to childhood respiratory health [24]. Environmental and dietary factors during pregnancy may affect development of infantile atopic dermatitis.

Nutrition and allergic diseases were studied in urban and rural communities from the South African Food Allergy cohort [25]. Allergy and sensitization rates are significantly higher in unselected urban South African toddlers than their rural counterparts. Risk and protective factors for allergy and atopy may differ between urban and rural settings. A study analyzed whether maternal consumption of selected Turkish fermented foods and other factors during pregnancy affect the development of atopic dermatitis during the first 2 years of life. Daily maternal intake of yogurt and diversity of consumed Turkish fermented foods during pregnancy may reduce the risk of atopic dermatitis [26].

There is a progressive increase of FcεRI expression across several peripheral blood mononuclear cell (PBMC) subsets which is associated with atopy and atopic asthma within school-aged children [27].

A multicenter, cross-sectional study examined the characteristics, quality of life and control of respiratory allergic diseases caused by house dust mites in Spain [28]. 296 patients were included; 60% had allergic rhinitis (mostly persistent moderate-to-severe) and 40% had rhinitis and asthma (mostly intermittent or mild-to-persistent). Patients with rhinitis had moderately reduced quality of life, which was significantly worse in adults than adolescents. The impact of asthma on quality of life was less pronounced than that of rhinitis.

The atopic syndrome consists of heterogeneous manifestations, in which multiple associated genetic loci have recently been identified. Immune dysregulation may play a role in the pathogenesis. Molecular clustering of genes related to the atopic syndrome was carried out [29]. Twenty-two (5.3%) genes were overlapping between the atopy-related genes (n = 160) and primary immunodeficiency disease-related genes (n = 278).

Eosinophilic disorders represent a group of pathologic conditions with highly heterogeneous pathophysiology and clinical presentation and variable prognosis, ranging from asymptomatic or mild, to severe and complex cases, with fatal outcome. A review of recent data was done from the literature regarding definition, classification and diagnosis criteria of eosinophilic diseases and to propose a revised and updated diagnostic algorithm useful in clinical practice [30].

Ataxia-telangiectasia is an autosomal recessive primary immunodeficiency disease that is caused by mutations in ataxia-telangiectasia mutated (ATM) gene encoding a serine/threonine protein kinase. Patients represent a broad range of clinical manifestations. Clinical features and molecular pathology were reviewed [31].

Several apps for asthma, rhinitis and chronic rhinosinusitis (CRS) were compared to propose some indications to users on their specificities and validation [32].

## Asthma

Histamine is an important immunomodulator influencing both the innate and adaptive immune system. In mice, bacterial secretion of histamine within the gut influences immune responses within the lungs [33].

In two independent microarray studies involving primary airway epithelial cells, the relative gene expression of TMEM178 decreases with the progression of asthma severity. TMEM178 encodes a negative regulator of NFATc1, which decreases with the progression of asthma severity [34]. The role of miRNAs in the pathogenesis and phenotypes of asthma is not fully elucidated. miR-146a has been previously shown to suppress inflammatory responses in different cells. In this study, it was found that reduced expression of miR-146a in human bronchial epithelial cells alters neutrophil migration and may contribute to the neutrophil phenotype of asthma [35].

The relationship between allergic sensitization during childhood and risk of developing asthma remains unclear [36]. Sensitization to multiple allergens at single time-points, increasing sIgE levels and SPT wheal sizes, and persistent sensitization during childhood were associated with increased risk of asthma at age 13, suggesting the use of quantitative and repetitive sensitization measurements when assessing risk of developing asthma.

Half of the adults with current asthma among the US National Health and Nutrition Examination Survey (NHANES) participants could be classified in more than one hypothesis-driven phenotype. A data-driven approach applied to the same subjects may allow a more useful classification compared to the hypothesis-driven one [37]. Both data- and hypothesis-driven approaches using clinical and physiological variables commonly used to characterize asthma are suboptimal to identify asthma phenotypes among adults from the general population. Further studies based on more comprehensive disease features are required to identify asthma phenotypes in population-based studies. In Southwestern Iran, a study assessed using the GA^2^LEN questionnaire showed that the current prevalence of asthma in adult population is 8.9% and asthma is often associated with chronic rhinosinusitis [38].

In the general population of children and young adults, daily short-acting, beta-agonist (SABA) sales are positively associated with Alternaria spore concentrations.. The association between daily SABA sales and temporal changes to *Alternaria* and *Aspergillus*-*Penicillium* in male children indicate that outdoor moulds contribute to asthma morbidity [39].

Blood eosinophil count and fractional exhaled nitric oxide (FeNO) concentration are established biomarkers in asthma, associated particularly with the risk of exacerbations. In an observational study using the Optimum Patient Care Research Database, eosinophils and FeNO are complementary and independent biomarkers of severe asthma exacerbations [40].

Over 30% of local allergic rhinitis patients self-report bronchial symptoms suggestive of asthma, but the relationship between the allergen exposure and the bronchial symptoms has not been studied. Bronchial asthma was triggered by house dust mites in patients with local allergic rhinitis [41]. The results suggest the existence of a new asthma phenotype (local allergic asthma) defined by absence of systemic atopy and positivity to bronchial provocation test with allergen.

There is wide variability in the response to inhaled corticosteroids (ICS) in asthma. While some of this heterogeneity of response is due to adherence and environmental causes, genetic variation also influences response to treatment and genetic markers may help guide treatment. A systematic review assessed the genetic associations of the response to inhaled corticosteroids in asthma [42]. To date, seven genome wide association studies (GWAS) examining ICS response have been published. Genes discovered in these GWAS (e.g. GLCCI1, FBXL7, T gene, ALLC, CMTR1) might play a direct or indirect role in asthma/treatment response pathways. Single nucleotide polymorphisms located in GLCCI1, NR3C1 and the 17q21 locus were positively replicated in independent populations. These studies might provide novel insights in the complex pathways underlying corticosteroids response in asthmatic patients.

In a Portuguese nationwide electronic prescription and dispensing database analysis, it was found that high oral corticosteroid exposure and overuse of short-acting beta-2-agonists were associated with insufficient prescribing of controller medication [43].

Evidence from observational comparative effectiveness research (CER) is ranked below that from randomized controlled trials in traditional evidence hierarchies. However, asthma observational CER studies represent an important complementary evidence source answering different research questions and are particularly valuable in guiding clinical decision making in real-life patients and practice settings. A Task Force was commissioned jointly by the European Academy of Allergy and Clinical Immunology (EAACI) and the Respiratory Effectiveness Group (REG) to develop a quality assessment tool for real-life observational research to identify high-quality real-life asthma studies that could be considered within future guideline development. The resulting REal Life EVidence AssessmeNT Tool (RELEVANT) was achieved through an extensive analysis of existing initiatives in this area. RELEVANT was then validated [44]. The development of RELEVANT, a novel quality assurance asset to rate observational comparative effectiveness research studies was reported in detail [45].

As shown in a cross-sectional study, psychosocial working conditions, asthma self-management at work and asthma morbidity are associated [46].

Most economies are struggling to deliver modern health care effectively. There is a need to support the transformation of the health care system into integrated care with organizational health literacy. Next-generation ARIA (Allergic Rhinitis and its Impact on Asthma) care pathways for rhinitis and asthma represent a model for multimorbid chronic diseases [47]. As an example for chronic disease care, MASK (Mobile Airways Sentinel NetworK), a new project of the ARIA (Allergic Rhinitis and its Impact on Asthma) initiative is proposing real-life ICPs centred around the patient with rhinitis, and using mHealth to monitor environmental exposure.

IL-33 plays a crucial role in initiation of type 2 immunity. A review addressed the current literature describing the role of IL-33 -responsive immune cells that may explain susceptibility to allergic sensitization at a young age and the association between genetic variants of IL-33 and asthma in humans [48].

## Allergic rhinitis

The roles of Histamine H4 receptor (H4R) have been characterized in T cell subsets, whereas the functional properties of H4R in monocytes remain unclear. H4R receptor regulates IL-6 and INF-gamma secretion in native monocytes from healthy subjects and patients with allergic rhinitis [49].

Links between multimorbidity of allergic diseases and allergen sensitization are still under debate, especially in adults. Multimorbidity is associated with polysensitization especially in children compared with adults in a Polish population cohort [50]. New insights into single disease patterns were found: bronchial asthma is the strongest risk factor for the development of multimorbidity in comparison with allergic rhinitis and atopic dermatitis.

There is little evidence on the incidence and characteristics of local allergic rhinitis in children. Most studies have included subjects with perennial rhinitis only, and results are based on the investigation of no more than three allergens per study. Local allergic rhinitis is present in a considerable proportion of children with chronic, difficult-to-treat rhinitis and no sensitization to aeroallergens, and therefore, the performance of nasal provocation test should be strongly considered in these cases. There were no distinct clinical characteristics between local allergic rhinitis, AR, and non-allergic rhinitis in children [51].

Clinically relevant effect of rupatadine 20 mg and 10 mg were examined in seasonal allergic rhinitis using a pooled responder analysis [52]. Data from 1470 patients were analyzed. A statistically higher proportion of patients reached the 50% and 75% response for total nasal and ocular symptom score in rupatadine groups compared to the placebo group across the visits. The time to achieve a proportion of responders was shorter in the rupatadine 20 mg group than in the rupatadine 10 mg and placebo groups for all the symptom scores. The number of patients who achieved a complete/near-to-complete response for both symptom scores was higher in rupatadine groups than in the placebo group, with higher proportions in the 20 mg group.

MASK has been awarded as a Good Practice of DG Santé (European Union) for digitally-enabled, integrated, person-centred care for rhinitis and asthma [53]. Lessons learnt by MASK include: (i) Adherence to treatment is the major problem of allergic diseases, (ii) Self-management strategies should be considerably expanded (behavioural), (iii) Change management is essential in allergic diseases, (iv) Education strategies should be reconsidered using a patient-centred approach and (v) Lessons learnt for allergic diseases can be expanded to chronic diseases.

## Rhinosinusitis and ENT

A 12-year follow-up study was carried out after endoscopic sinus surgery in 47 patients with chronic rhinosinusitis with nasal polyposis [54]. This long-term cohort study, investigating the outcome after surgery, showed that despite the low number of patients, 78.9% of patients were subject to recurrence of the disease and 36.8% to revision surgery over a 12-year period.

Rhinovirus A and C infections are important contributors to asthma induction and exacerbations. No data exists on the interaction of local immune responses in rhinovirus infection. A study aimed to determine the tonsillar immune responses according to rhinovirus A, B and C infections. Rhinovirus species are associated differently with clinical characteristics and tonsillar cytokine responses [55].

## Urticaria and angioedema

Exploratory literature search has been done for reports that have evidence of organ-specific dysfunction in chronic urticaria. This search revealed some evidence of systemic effects of chronic urticaria in cardiac, respiratory, gastrointestinal, central nervous and musculo-skeletal systems that might justify prospective observational studies [56].

In line with the recent guideline recommendation for the use of second generation H1 antihistamines in children with chronic urticaria, bilastine was found to have a good safety and tolerability profile [57].

Biomarkers and clinical characteristics of autoimmune chronic spontaneous urticaria (aiCSU) were examined in the PURIST Study [58]. aiCSU is a relatively small but immunologically distinct subtype of CSU that cannot be identified by routine clinical parameters. Inclusion of basophil histamine release assay or basophil activation test in the diagnostic workup of urticaria patients may aid identification of aiCSU patients, who may have a different prognosis and benefit from specific management.

The Icatibant Outcome Survey allowed to identify the phenotype of patients with hereditary angioedema type I/II [59]. Elderly patients had significantly more comorbidities and were receiving a greater number of concomitant medications. Antihistamines are the most prescribed therapy in recurrent idiopathic angioedema, yet little is known about their efficacy. In 120 patients, a high incidence (36%) of antihistamine refractory cases was observed, patients on antihistamine prophylaxis suffered from 1 or more angioedema attacks per month [60].

Psychiatric comorbidity in chronic urticaria patients was examined in a systematic review and meta-analysis [61]. Patients with CU frequently experience psychiatric disorders. This highlights the need for a multidisciplinary therapeutic approach involving prompt recognition and management of any potential psychiatric disorder in addition to urticaria treatment. Further studies are needed to assess whether psychiatric disorders coexist with CU independently or follow urticaria onset and whether any psychological or psychiatric intervention may help in CU control.

The recently described plasminogen gene mutation p.Lys330Glu was described in a family from Northern Germany with hereditary angioedema [62].

In an observational study, increased platelet activating factor levels in chronic spontaneous urticaria predicted refractoriness to antihistamine treatment [63].

## Atopic dermatitis

Many skin and mucosal inflammatory disorders, such as atopic dermatitis, have been associated with an impaired epithelial barrier function, which allows allergens, pollutants, or microbes to enter the tissue and activate the immune response. The aim of this study was to establish a method to directly assess in vivo the epidermal barrier function by electrical impedance spectroscopy. Direct assessment of skin epithelial barrier can be observed by electrical impedance spectroscopy [64]. There is a transient epidermal barrier deficiency and lowered allergic threshold in filaggrin–hornerin (FlgHrnr(−/−)) double-deficient mice [65].

House dust mite sensitisation is associated with atopic dermatitis in Brunei [66].

Atopic dermatitis (AD) is a complex heterogeneous chronic inflammatory skin disease. Specific IgE antibodies against autoantigens have been observed in a subgroup of AD patients, however, little is known about IgG-auto-reactivity in AD. The antimicrobial protein S100A12 identified as a potential autoantigen in a subgroup of atopic dermatitis patients [67].

The prevalence of food hypersensitivity in relation to IgE sensitisation to common food allergens was studied in the general adult population in West Sweden [68]. The prevalence of self-reported food hypersensitivity in West Sweden is rising. The correspondence between self-reported symptoms and IgE-sensitisation to foods is generally poor, except for hazelnut and almond which exhibit moderate or fair correlation.

## Other skin diseases

In cold medicine-related Stevens-Johnson syndrome, in addition to the Japanese, Korean and Indian populations, Thai cases with cold-related Stevens-Johnson syndrome and severe ocular complications were significantly associated with IKZF1 single nucleotide polymorphism (SNPs) [69].

Scabies is a common contagious skin disease caused by an infestation of the skin by *Sarcoptes scabiei*. A hallmark symptom of scabies is severe itch which may be associated with the generation of a pruritogenic cytokine, interleukin (IL)-31 generated by M2 macrophages in ordinary scabies lesions. This IL-31 induction was induced by overexpression of thymic stromal lymphopoietin and periostin [70].

Cosmetics and skin care products for neonates and infants are considered as ‘‘hypoallergenic’’, “tested” or ‘‘safe’’. Nevertheless, the prevalence of haptens in these products is a matter of concern, since allergic contact dermatitis in children is becoming more prevalent [71]. 85% of products for children labeled as hypoallergenic/dermatologically tested/safe for children contained haptens.

## Food allergy

Vagus nerve stimulation dampens intestinal inflammation in a murine model of experimental food allergy [72].

Outcome of oral immunotherapy for persistent cow’s milk allergy was reported from 11 years of experience in Finland [73]. 296 children aged 5 years or older with IgE-mediated cow’s milk allergy (CMA) started milk oral immunotherapy (OIT). More than half of the patients were able to maintain the targeted milk dose in their daily diet. Baseline milk sIgE level and reactivity during the early treatment stage strongly predicted the long-term outcome and safety of milk OIT.

Tolerance development rates differ between food allergies. Almost all previous studies have not used the gold standard method, the double-blind, placebo-controlled food challenge (DBPCFC), which may affect the reported prevalence rates. Little is known about the association of the eliciting dose (ED) obtained during the initial DBPCFC with later tolerance development. In a retrospective study in children who had a positive DBPCFC to either peanut, milk or egg, and at least one follow-up food challenge (open or DBPCFC) with the same food. Approximately 1 out of 4 children with DBPCFC confirmed peanut allergy developed tolerance, compared to more than half of the children with milk or egg allergy, respectively. Tolerance development in peanut and milk allergy is significantly associated with ED at initial DBPCFC [74].

The clinical threshold in wheat-dependent, exercise-induced anaphylaxis seems to be lowered in patients on a wheat-free diet, whereas the opposite is seen in patients on a regular wheat intake. A recommendation of wheat consumption, if considered safe to the patient based on case-history and challenge results could be advised [75]. Sesame oleosins are minor allergens [76]. Lipid Transfer Protein allergy was characterized in the United Kingdom and compared with a matched Italian cohort [77]. Native UK subjects with LTP allergy are not dissimilar to those with LTP allergy in southern Europe. A simple tool for diagnosis of milk- and wheat protein drinks for double blind placebo controlled food challenge in adults was proposed [78].

The characterization of a subgroup of non-type 2 asthma with cow’s milk hypersensitivity was done in young subjects (up to 35 years of age) [79]. It was found a poor asthma-related quality of life, airway hyperresponsiveness, and clinically relevant non-type 2 inflammation. MMP-1 was elevated in this group, which deserves further studies.

Deficits in regulatory T-cell (Treg) number and function at birth have been linked with subsequent allergic disease. However, longitudinal studies that account for relevant perinatal factors are required. A study aimed to investigate the relationship between perinatal factors, naive Treg (nTreg) over the first postnatal year and development of food allergy [80]. In a birth cohort (n = 1074), infants that developed food allergy had decreased naïve Treg at birth, and the labour-associated decrease in naïve Treg at birth was more evident among infants with subsequent food allergy.

Food allergy diagnosis is difficult and in Finland a structured intervention plan including component-resolved diagnostics helped reducing the burden of food allergy among school-aged children [81].

A randomized controlled trial was carried out in specific synbiotic-containing amino acid-based formula in dietary management of cow’s milk allergy (CMA) [82]. Fecal percentages of bifidobacteria and *Eubacterium rectale*/*Clostridium coccoides* group were assessed. The trial included 35 test subjects, 36 controls, and 51 in the healthy reference group. At week 26, median percentages of bifidobacteria were significantly higher in test than control [47.0% vs. 11.8% (p < 0.001)]. Use of dermatological medication and reported ear infections were lower in test versus control. The same specific synbiotic-containing amino acid-based formula restored gut microbiota in non-IgE mediated cow’s milk allergic infants [83]. The formula effectively modulated the gut microbiota and its metabolic activity in non-IgE mediated CMA infants bringing it close to a healthy breastfed profile.

An update to the Milk Allergy in Primary Care guideline was proposed [84]. The Milk Allergy in Primary (MAP) Care guideline was first published in 2013 in this journal. In 2018, the guidelines were criticised for 3 distinct reasons: promoting the overdiagnosis of CMA, negatively impacting breastfeeding and the possibility of industry influence on the guidelines. The authors address these criticisms using available evidence and, in the context of this and in consultation with patient groups, members of the General Practice Infant Feeding Network and other infant feeding healthcare leads, have collaboratively produced updated algorithms and an information leaflet to support breastfeeding. We believe the updated International Milk Allergy in Primary Care (iMAP) is now closer to its original aim of facilitating early and accurate diagnosis of CMA, whilst minimising, as far as possible, any concerns around overdiagnosis or a risk to breastfeeding rates. We continue to welcome open and constructive engagement about how best to achieve these aims to provide evidence-based, practical guidelines for the primary care practitioner.

Health-related quality of life worsens by school age amongst children with food allergy [85].

## Anaphylaxis, drug reactions

Selective reactions to clavulanic acid (CLV) account for around 30% of immediate reactions after administration of amoxicillin-CLV. Currently, no immunoassay is available for detecting specific IgE to CLV, and its specific recognition in patients with immediate reactions has only been demonstrated by basophil activation testing, however with suboptimal sensitivity [86]. Expression of the Tim3-galectin-9 axis is altered in drug-induced maculopapular exanthema [87]. Skin tests are important in children with beta-lactam hypersensitivity, but may be reduced in number [88].

Risk factors for severe systemic sting reactions in wasp (*Vespula* spp.) and honeybee (*Apis mellifera*) venom were examined in 480 allergic patients [89]. The clinical history is essential for the allergological workup and therapeutic decision on Hymenoptera venom allergies. A short latency time and the absence of skin symptoms are indicators for severe systemic sting reactions, followed by the patient’s age and baseline serum tryptase levels. Large local reaction to Hymenoptera stings is usually defined as a swelling > 10 cm which lasts longer than 24 h, sometimes associated with erythema, pruritus and blisters. Currently, the risk of subsequent systemic reactions after re-stings is considered low (2–15%). Therefore, a diagnostic workup in case of large local reaction is often judged unnecessary, as well as adrenaline auto-injector and venom immunotherapy prescription. Outcome of large local reactions during re-stings in real-life were studied [90]. Out of 225 subjects, 24% had a systemic reaction. Systemic reactions, after a previous large local reaction, occur more frequently than that reported by literature. After analysing the predictive role of large local reactions for systemic reactions, we demonstrated that an accurate diagnostic workup may be considered, particularly skin tests.

In wheat-dependent exercise-induced anaphylaxis (WDEIA), cofactors such as exercise, acetylsalicylic acid (ASA), alcohol or unfavorable climatic conditions are required to elicit a reaction to wheat products. However, they do not increase highly individual gliadin absorption in healthy volunteers [91].

The correct classification of an adverse drug reaction (ADR) as allergy (immunological) or intolerance (non-immunological) has important clinical implications. [92] The aim of this study was to examine the ability of health professionals to discriminate between allergy and intolerance, classify the severity of the ADR and degree of contraindication. Overall 59.0% (SD 28.9) correctly categorised the cases, 60.8% (SD 16.8) classified the severity correct, and less than half (44.7%, SD 28.6) correctly identified the level of contraindication. Strategies are required to strictly avoid re-exposure of patients to drugs which carry an increased risk of inducing a dangerous reaction, whilst minimising the avoidance of drugs which are of minimal risk or allowing the use of low-risk drugs where the benefits may be significant.

A study evaluated adherence to adrenaline autoinjector prescriptions in a cohort of well-characterized anaphylaxis patients [93]. The overall retrieval rate was 76% with the highest rate in patients with severe anaphylaxis. Special attention is needed in patients with unknown elicitors and in young adults, comprising the largest proportion of non-adherent patients. The basophil activation caused by transgenic rice seeds expressing whole T cell epitopes of the major Japanese cedar pollen allergens was evaluated and may represent an interesting model to screen T cell epitopes [94].

A EAACI drug allergy interest group surveyed on how European allergy specialists deal with beta-lactam allergy [95]. A significant heterogeneity exists in current practice not only among countries, but also among centres within the same country. This suggests the need to re-evaluate, update and standardize protocols on the management of patients with suspected beta-lactam allergy.

## Immunotherapy (AIT)

EAACI has developed a clinical practice guideline providing evidence-based recommendations for the use of house dust mites (HDM) AIT as add-on treatment for HDM-driven allergic asthma. The guideline was developed by a multi-disciplinary working group using the Grading of Recommendations Assessment, Development and Evaluation (GRADE) approach. HDM AIT was separately evaluated by route of administration and children and adults: subcutaneous (SCIT) and sublingual AIT (SLIT), drops, and tablets. Recommendations were formulated for each. The important prerequisites for successful treatment with HDM AIT are (a) selection of patients most likely to respond to AIT and (b) use of allergen extracts and desensitization protocols of proven efficacy. An algorithm for diagnosing allergic asthma and for introducing AIT as add-on to asthma treatment is provided as a tool for the clinician in the decision-making process [96].

IL-4 receptor alpha blockade prevents sensitization and alters acute and long-lasting effects of allergen-specific immunotherapy of murine allergic asthma [97].

Immunomodulatory effects of sublingual immunotherapy on systemic and mucosal mediators in allergic children are largely unexplored. *Phleum pratense* sublingual immunotherapy in grass pollen allergic children modulates the immune response in the oral mucosa as well as systemically-by increasing Th1-responses, decreasing Th2-responses, and inducing immunoregulatory responses-all signs of tolerance induction [98].

A global approach has been proposed for the contraindications of AIT [99]. An extended review of the literature regarding contraindications to AIT for respiratory allergy and venom hypersensitivity was performed. Furthermore, Societies and Academies registered in the World Allergy Organization and EAACI databases were asked for additional information. Only AIT guidelines published under official auspices were included. A large heterogeneity among the various recommendations on contraindications was registered. Common contraindications to most of the guidelines were: lack of adherence, pregnancy before the start of AIT, the use of beta-blockers, certain age groups, uncontrolled asthma, autoimmune diseases and malignancies.

Efficacy of house dust mite sublingual tablet in the treatment of allergic rhinoconjunctivitis was studied in a randomized trial in a pediatric population [100]. The HDM tablet significantly improved symptoms of HDM-induced perennial AR and was associated with a significant immunological response. The safety profile in pediatric patients was consistent with that in adults, with no new safety concerns.

A systematic review and meta-analysis were carried out in subcutaneous immunotherapy with depigmented-polymerized allergen extracts [101]. Compared to placebo, AIT with depigmented-polymerized allergen extracts is effective in patients with pollen- or HDM-induced rhinoconjunctivitis with or without allergic asthma and improves health-related quality of life. It does not differ significantly in safety and tolerability.

Two grass pollen tablets commercially available for allergy immunotherapy display different IgE epitope repertoires [102]. Compared to the 1-grass tablet, the 5-grass tablet generally covers better the sensitization profiles of European patients, especially patients from Southern Europe, in principle less exposed to pollen from Timothy than from other grasses.

Data regarding clinical relevance of HDM components over allergen immunotherapy (AIT) for allergic rhinitis (AR) are lacking. 18 adult AR patients receiving HDM-AIT for 52 weeks were followed up to assess serum levels of sIgE and sIgG4 to HDM components. The study showed that Der p1, p2, p23, Der f1 and f2, are important sensitizing components of HDM, of which Der p1 appears to be the most clinically relevant allergenic component for effective AIT [103].

Japanese cedar (JC) pollinosis is a serious type I allergic disease in Japan. Although subcutaneous immunotherapy and sublingual immunotherapy have been applied to treat JC pollinosis, high doses of allergens may cause IgE-mediated allergic reactions. The transgenic rice seeds that contain genetically modified Cry j 1 and Cry j 2, the two major allergens of JC pollen, have been developed as candidates for oral immunotherapy [94]. In HDM, Der p 1, 2, 5, 7, 21, and 23 are the most important allergens. A study aimed to define hypoallergenic peptides derived from the sequences of the six allergens and to use the peptides and the complete allergens to study antibody, T cell, and cytokine responses in sensitized and nonsensitized subjects [104]. HDM allergic patients showed IgE reactivity only to complete allergens. IgG antibodies in HDM-allergic patients recognize peptide epitopes which are different from the epitopes recognized by IgE. This may explain why naturally occurring allergen-specific IgG antibodies do not protect against IgE-mediated allergic inflammation.

## Political agenda

EAACI organized the first European Strategic Forum on Allergic Diseases and Asthma. The main aim was to bring together all relevant stakeholders and decision-makers in the field of allergy, asthma and clinical Immunology around an open debate on contemporary challenges and potential solutions for the next decade. The Strategic Forum was an upscaling of the EAACI White Paper aiming to integrate the Academy’s output with the perspective offered by EAACI’s partners. An open debate with a special focus on drug development and biomedical engineering, big data and information technology and allergic diseases and asthma in the context of environmental health concluded that connecting science with the transformation of care and a joint agreement between all partners on priorities and needs are essential to ensure a better management of allergic diseases and asthma in the advent of precision medicine together with global access to innovative and affordable diagnostics and therapeutics [105].

Allergic diseases and asthma represent over 25% of the European population and cause a very high burden. Strategies for early diagnosis, prevention and control need to be anchored on a strong political agenda to implement the results of the research into practice. One important political activity at the EU level was reported: the Vilnius Declaration on chronic respiratory diseases held in the Lithuanian Parliament March 2019 [106].

The Nature Step to Respiratory Health was the overarching theme of the 12th General Meeting of the Global Alliance against Chronic Respiratory Diseases (GARD) in Helsinki, August 2018. New approaches are needed to improve respiratory health and reduce premature mortality of chronic diseases by 30% till 2030 (UN Sustainable Development Goals, SDGs). Planetary health is defined as the health of human civilization and the state of the natural systems on which it depends [107]. “Helsinki by Nature” reported this meeting and proposed the nature steps to respiratory health.

The BelSACI-Abeforcal-EUFOREA statement proposed a stepwise approach towards adoption of allergen immunotherapy for allergic rhinitis and asthma patients in daily practice in Belgium [108].

## Data Availability

Not applicable.

## Abbreviations

AD: Atopic dermatitis
ADR: Adverse drug reaction
aiCSU: Autoimmune chronic spontaneous urticaria
AIRWAYS ICPs: Integrated care pathways for airway diseases
AIT: Allergen immunotherapy
AR: Allergic rhinitis
ARIA: Allergic Rhinitis and its Impact on Asthma
BelSACI: Belgian Society for Allergy and Clinical Immunology
CER: Comparative effectiveness research
CHRODIS: Addressing Chronic Diseases and Healthy Ageing across the Life Cycle
CLV: Clavulonic acid
CMA: Cow’s milk allergy
COPD: Chronic obstructive pulmonary disease
CRS: Chronic rhinosinusitis
Cry j: *Cryptomeria japonica*
CU: Chronic urticaria
DB-PC-FC: Double-blind, placebo-controlled food challenge (DBPCFC)
Der p: *Dermatophagoides pteronyssinus*
EAACI: European Academy of Allergy and Clinical Immunology
ED: Eliciting dose
EMT: Epithelial-to-mesenchymal transition
ENT: Ear Nose and Throat
EPOS: European position paper on rhinosinusitis
EUFOREA: European Forum for Research and Education in Allergy and Airway Diseases
FeNO: Fractional exhaled nitric oxide
GA^2^LEN: Global Allergy and Asthma European Network
GARD: Global Alliance against Chronic Respiratory Diseases
GWAS: Genome-wide association study
H4R: Histamine H4
HDM: House dust mite
ICS: Inhaled corticosteroids
IKZF1: Ikaros Zinc finger protein 1
iMAP: Milk Allergy in Primary Care guideline
INCS: Intra-nasal corticosteroid
LC-PUFAs: Long-chain polyunsaturated fatty acids
LTP: Lipid Transfer Protein
MASK: Mobile Airways Sentinel NetworK
miR: Micro-RNA
MMP-1: Matrix metallo protease 1
NFATc1: Nuclear factor of activated T-cells, cytoplasmic 1
PASTURE: Protection Against Allergy: Study in Rural Environments
PURIST: Profiling Urticaria for the Identification of Subtypes
RELEVANT: REal Life EVidence AssessmeNT Tool
SABA: Short-acting, beta-agonist
SPT: Skin prick test
ST2: IL-33 receptor
TMEM178: Transmembrane protein 178
Treg: Regulatory T cell
